# Apoptotic Janus-faced mycotoxins against thoracal and breast metastases

**DOI:** 10.1007/s10495-023-01837-1

**Published:** 2023-04-13

**Authors:** Gaspar Banfalvi

**Affiliations:** Department of Molecular Biotechnology and Microbiology and Department of Physiology, University of Debrecen, 1 Egyetem Square, Life Sciences Building 1.102, Debrecen, 4010 Hungary

**Keywords:** Carcinogens, Antitumour agents, Double-edged molecules, Metastatic tumour models, Abdominal tumours, Metastatic spread to PTNS, Prevention of breast cancer

## Abstract

Abdominal organs (liver, kidney, spleen) are frequent targets of cancer cell invasion but their primary tumours are less known for their metastatic potential to other organs e.g. to the breast. Despite the known connection of the pathogenesis from breast cancer to liver metastasis, the study of the spread in the opposite direction has been neglected. The notion that breast cancer could be a metastasis besides being a primary tumour is based on rodents’ tumour models upon implantation of tumour cells under the capsule of the kidney or under the Glisson’s capsule of the liver of rats and mice. Tumour cells develop into a primary tumour at the site of subcutaneous implantation. The metastatic process starts with peripheral disruptions of blood vessels near the surface of primary tumours. Tumour cells released into the abdomen cross the apertures of the diaphragm, enter the thoracal lymph nodes and accumulate in parathymic lymph nodes. Abdominal colloidal carbon particles injected into the abdomen faithfully mimicked the migration of tumour cells and deposited in parathymic lymph nodes (PTNs). An explanation is provided why the connection between abdominal tumours and mammary tumours escaped attention, notably, parathymic lymph nodes in humans were referred to as internal mammary or parasternal lymph nodes. The apoptotic effect of Janus-faced cytotoxins is suggested to provide a new approach against the spread of abdominal primary tumours, and metastatic development.

## Introduction

### Janus-faced molecules

Molecules acting as double-edged swords, also referred to as Janus-faced molecules possess both beneficial and harmful biological effects. Due to their side effects, these compounds were not preferred in medical practice, which does not mean that their health benefits could not be utilized. A known double-edged sword is resveratrol, found in many plant species that belong to the polyphenol stilbenoids group containing two phenyl rings attached through an ethylene bridge [1]. The polyphenol resveratrol contributes to the potential health benefits attributed to moderate wine consumption known as the “French Paradox” [2]. The term Janus-faced molecules also refers to the harmful genotoxic (teratogenic, carcinogenic, mutagenic) effects [3, 4]. on the one hand and the cytocidal and antineoplastic properties on the other hand [5–7]. An enormous source of Janus-faced molecules is represented by the secondary metabolites of fungal mycotoxins affecting eukaryotic cell growth, structurally and functionally contributing to the multitude of their classifications. The”double-edged sword” effect suggested the utilization of mycotoxins against plant pathogenic fungi causing life-threatening human diseases [8].

A classical example of a double-edged sword is cholesterol. High levels of cholesterol cause diabetes, high blood pressure, vascular and other diseases. Due to the low solubility, its high level may cause coronary heart attack that can lead to death. The amphipathic property and the shape of the cholesterol molecule make it suitable as a membrane insert [9]. Cholesterol is providing both mechanical stiffness and elasticity to cells by the polar head groups of cholesterol oriented toward the polar heads of phospholipids. The relationship of cholesterol to anticancer drugs revealed that antitumour agents selectively inhibit cholesterol biosynthetic enzymes [9]. Prokaryotic cells do not contain cholesterol but the related pentacyclic triterpenoids collectively termed hopanes. The most promising hopane is celastrol, a triterpene that exhibits significant anticancer activities [10].

Another double-edged sword is nitric oxide (NO), one of the major air pollutants but also a gaseous signalling molecule playing an important role as a key vertebrate biological messenger [11–13]. Nitroso compounds, among them polycyclic aromatic hydrocarbons and heterocyclic amines formed during food preservation, and cooking have beneficial anticarcinogenic activities [14]. The chemical carcinogenesis is also inhibited by allyl sulfur compounds such as diallyl sulfide [15, 16], isolated from garlic. The double-edged curcumin is known for its cancer-preventive effect [17].

Three types of antifungal agents have been reviewed recently, including double-edged mycotoxins, aminoglycoside molecules as double-edged swords, among them the most efficient minor fraction gentamicin B1, and the classical antifungal agents. Particular attention was given to the antifungal activities of these compounds in comparison with other classical antifungal drugs (amphotericin B, clotrimazole, nystatin, and griseofulvin). The poisonous mycotoxins ubiquitous in nature are produced by various fungi species whose occurrence in the food chain is inevitable. Mycotoxins pose a serious problem on a global scale [18, 19] since toxic human exposure to mycotoxins is commonly due to food and feed contamination [20, 21]. The double-edged sword effect of antineoplastic mycotoxins has been reviewed concerning selected mycotoxins that affect eukaryotic cells with a broad range of structural and functional groups, allowing different types of contributing classifications [22].

### IARC categories of Janus-faced mycotoxins

The anticancer properties of double-edged compounds could be utilized for the benefit of patients when their survival is more important than the mutagenic side effects caused by these compounds. To decide which fungal toxins could be utilized alone or in combination with other antimetastatic agents, the side effects have to be clarified. The International Agency for Research on Cancer (IARC, Lyon, France, 2021) distinguished three categories. Group 1) IARC compounds are carcinogenic to humans, and Group 2 compounds are probable carcinogens. Group 2A is probably carcinogenic to humans, group 2B is reasonably anticipated to be a human carcinogen. Group 3 is lacking carcinogenic properties, possesses tolerable side effects, and more importantly is among the candidates for antimetastatic agents. From the structure–function relationship of mycotoxins, only general conclusions have been drawn. Ring structures that contain heteroatoms, functional groups and the cumulative presence of oxygen atoms contribute to the oxidative stress and cytotoxicity of mycotoxins. The preselection of mycotoxins for medical treatment excludes category 1 and 2 IARC carcinogens, and only the IARC category 3 compounds have been considered as potential antitumour agents against the metastatic spread of abdominal tumours. By analogy in humans, a similar tumour spread could take place that could be prevented by apoptotic antineoplastic double-edged sword molecules. In conformity with this strategy in mind, the focus is placed on those mycotoxins and their precursors that besides being highly cytotoxic, attest to antitumour properties. No doubt that some of them will not only be considered antimetastatic agents but used to prevent patients diagnosed of having thoracal and mammary lymph node tumours. The cytotoxic side effect could induce mutagenesis that could develop into scondary tumours but later when the Janus-faced molecules already exerted their beneficial antineoplastic effects.

### Double-edged antitumour mycotoxins

**Fig. 1:**
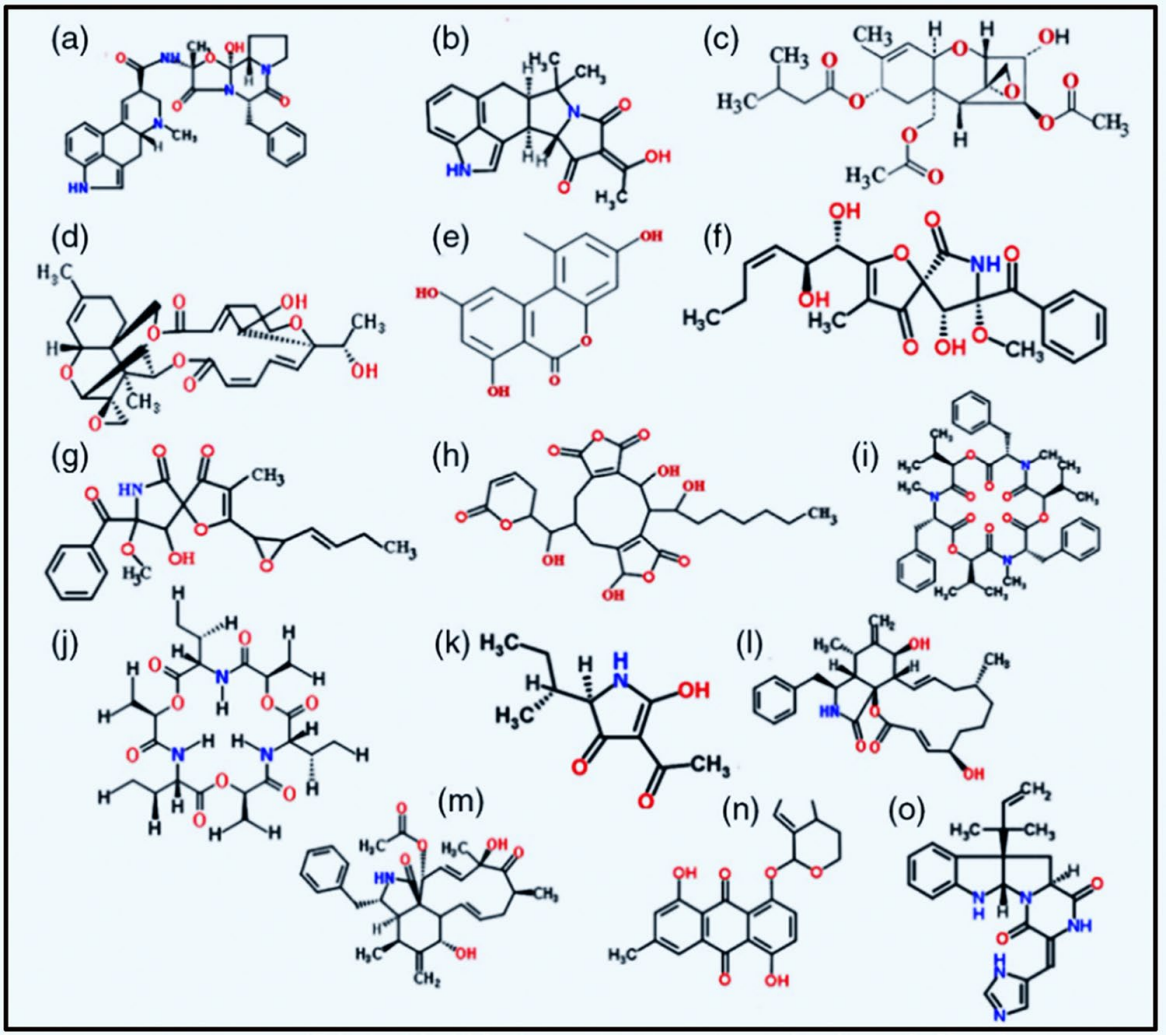
Chemical structures of selected mycotoxins with strong toxic effects and anticancer potential. **a** Ergotamine, **b** cyclopiazonic acid, **c** T-2 toxin, **d** satratoxin H, **e** alternariol,**f** pseurotin, **g** synerazol, **h** rubratoxin, **i** beauvericin, **j** enniatin, **k** tenuazonic acid,**l** cytochalasin B, **m** cytochalasin C, **n** MT81, and **o** roquefortine of beneficial low toxicity. Permitted by [22]

Different types of antifungal agents have been reviewed recently, including the’double-edged’ mycotoxins, and aminoglycoside molecules as double-edged swords, among them gentamicin B1, the most efficient minor fraction of the gentamicin family. Particular attention was given to the antifungal activities, compared to classical antifungal drugs such as amphotericin B, clotrimazole, nystatin, and griseofulvin [23]. Fungi contain structurally diverse metabolites. In the past, it was thought that medicine was present in grass and wood. In the future, we have to include fungi as useful sources of pharmaceuticals. The double-edged sword effect of those antineoplastic mycotoxins has been reviewed that affect eukaryotic cells and contain a broad range of structural and functional groups, contributing to their classification. Without striving for completeness only those double-edged mycotoxins will be dealt with that possess besides the strong cytotoxic activity the beneficial anticancer potential (Fig. 1). The strong toxic effects and the beneficial anticancer potential of the mycotoxins captured our attention [22].

**Fig. 2:**
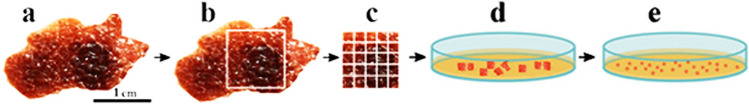
Establishment of rat hepatocarcinoma tumour cell line (HeDe). Under strictly sterile conditions **a** Hepatocarcinoma tumour of the rat was surgically removed, **b** designated part of the tumour (boxed), **c** was minced into 2 × 2x2 mm pieces, **d** and digested with elastase medium, filtered through sterile gauze, suspended in RPMI 1640 medium. After overnight incubation at 37 °C in a CO_2_ incubator, the non-adherent cells were discarded and the adherent cells were subcultured. **e** After 20 subcultures the new hepatocarcinoma He/De cell line was established. The same procedure was applied when other (Ne/De, My1/De and My2/De) cell lines were isolated [40]

The beneficial apoptotic effect of cytotoxicity is one of the potentials that can be utilized against the spread of cancer. Antifungal cytotoxins have been reluctantly applied against fungal infections as they raise ethical issues due to their mutagenic impact. Nevertheless, their utilization deserves consideration and could be useful not only for patients who suffer from fungal infections. This study suggests the extension of the application of Janus–faced molecules against breast cancer and mammary metastasis. Breast cancer patients are normally older than their fertility age. The short-term cytotoxicity of cancer treatment is likely to be favoured over the long-term mutagenicity. The short-term and beneficial anticancer treatment could induce secondary tumour development but years later of treatment when primary cancer or metastasis has been eradicated. Primary tumours were used to establish tumour cell lines that allowed to follow the development of metastasis in different organs and lymph nodes in the body of rodents (rats and mice). The establishment of tumour cell lines is unavoidable to secure the implantation of an exact number of cells and follow reproducibly the temporal aspects of the metastatic development. The preparation of tumour cell lines is schematically shown in Fig. 2.

We did not carry out research that involved human subjects. Regarding the ethical issue of animal experiments: rats and mice were kept in a conventional laboratory environment and fed on a semi-synthetic diet (Charles River Mo, Kft, Godollo, Hungary) and tap water ad libitum. The conditions under which animal experiments were performed are consistent with prevailing standards in the UK. Animals received human care to the criteria outlined in the Guide for the Care and Use of Laboratory Animals authorised by the Ethical Committee for Animal Research, University of Debrecen, Hungary (permission # 22). All experiments conformed to the European Convention for the protection of Vertebrate Animals used for Experimental and Other Scientific Purposes, 2010.

The majority of the anticancer Janus-faced mycotoxins belong to the trichothecene analogues [22]. Mycotoxins belonging to the large family of trichothecenes are produced by moulds such as *Fusarium, Trichoderma, Trichthecium, Myrothecium, Trichothecium, Cephalosporium, Ventrici Monosporium.* The most important structural elements of trichothecenes are the 12,13-epoxy rings responsible for the major toxicity, the presence of hydroxyl and acetyl groups and the side chains [24]. The trichothecenes induce apoptotic cell death [25]. Trichothecenes, primarily anguidine anal have already been subjected to drug trials [26–29]. Mycotoxins with antitumour activities have been found in different fungal cell lines [30, 31]. The antitumour activities among others the following mycotoxins deserve further investigation [22]:

- mycophenolic acid, penicillic acid, 5-methoxysterigmatocystin

- anguidine analog scirpenol, di- and triacetoxyscirpenols

- T-2 toxin and related trichothecenes

- cytochalasin B

- patulin

- hydromytoxin B

- 16-hydroxyroridin E

- tenuazoic acid

- betaacetoxyscirpendiol

- gliotoxin

- fluorinated pseurotin A

- synerazol

- rubratoxin B

- beauvericin

- the macrocyclic trichothecenes verrucarin A and roridin A

- leuteoskyrin, a hydroxyanthraquinonederivativee related to MT81.

The efficient apoptotic effect of cytotoxicity seems to be suitable to be utilized against the spread of cancer. Despite some secondary metabolites reaching clinical trial phase II and more mycotoxin derivatives being expected to enter human clinical trials shortly, no fungi-derived agent has been approved as a cancer drug so far [31, 32]. Despite the tremendous effort being aimed at the identification of fungal metabolites, the lack of breakthrough is probably related to the mutagenicity of mycotoxins. Ethical issues of consideration deserve careful selection, restructuring of selected mycotoxins, replacements and testing of the role of functional groups. The following rule applies to all double-edge compounds: insufficient knowledge requires caution before the anticancer and antimetastatic potentials can be realistically estimated. This study suggests that after selection and fine-tuning the antiapoptotic effect Janus–faced molecules could be used against the spread of metastasis. The short-term and drastic anticancer treatment of mycotoxins is favoured to reduce the risk of long-term mutagenicity side effects. The warning is repeated: the mutagenic side effect may induce secondary tumour development at a later time but the immediate antitumour treatment might be more important. Tumorigenic mycotoxins are not discussed here as they are not to be involved in medical treatment. Several structural features of tumorigenic mycotoxins contribute to their IARC classification.

### Metastatic animal models

Due to the complexity of animal tumours, it is impossible to use a single solution and requires selection and application among models. The chicken chorioallantois-membrane model is among the oldest ones to study metastasis [33] but its simplicity is contrasted by its suitability questioning whether the evolutionary gap between birds and mammalians can be bridged. The metastatic tumour model is not to be confused with the metastatic hypothesis the bests known of which is the”seed and soil” hypothesis. This assumption suggested that tumour cells similarly to seeds of plants are carried in all directions but grow only at locations where conditions are suitable for growth [34]. The”seed and soil” theory was challenged [35] and revisited [36]. Metastatic tumour models that have been favoured are syngeneic and xenograft designs, which are commonly and successfully used to model the metastatic processes associated with various types of cancers in animals. We deal first with the metastatic tumour models and summarize the favoured metastatic theory at the end of the review.

The administration of tumour cells is prohibited in man, thus we have developed rat and murine models from N-dimethylnitrosamine–induced tumours, namely from mesenchymal nephroblastoma and hepatocarcinoma [37]. The major advantage of using tumour cell lines over tumour slices is that they were applied earlier for the transplantation of tumour cells under the subrenal capsule of the kidney or Glisson’s capsule of the liver of rodents (rats, mice) securing the implantation of an exact number of cells. The accurate number of transplantation allowed to follow the reproducible temporal progression of the metastatic process.

Secondary tumour growth (metastasis) was measurable after 8 days of tumour cell implantation under the subrenal capsule or in the Glisson’s pocket of the liver of rats resulting in secondary tumours in the thoracal lymph nodes primarily in parathymic lymph nodes (PTNs). The initialhaematogenouss growth of the primary tumour is characterized by its hypoxic conditions and apoptosis inside the tumour, growth outward and disruption of the peripheral blood vessels due to the lagging angiogenesis and release of blood and tumour cells into the peritoneal cavity. The lymphatic part of the tumour spread starts when the increased volume of ascitic fluid and tumour cell accumulation in the peritoneal transudate forces tumour cells to cross the transdiaphragmatic pores and drain tumour cells into the thoracal lymphatic vessels and lymph nodes. Metastatic cells accumulate in the sentinel lymph nodes primarily in the internal mammary and parathymic lymph nodes (IMNs, PTNs). The accumulation of tumour cells could increase the volume of PTNs up to 70-times and their weight from 10 to 700 mg. India ink was implanted under the kidney capsule of rats and mice to mimic the lymphatic spread of colloidal particles in the thoracal lymph duct and nodes. The metastatic process was mimicked by the implantation and appearance of India ink colloidal particles in PTNs and confirmed the general mechanism from abdominal primary tumour growth through blood circulation and lymphatic development of metastasis in the thorax.

**Fig. 3:**
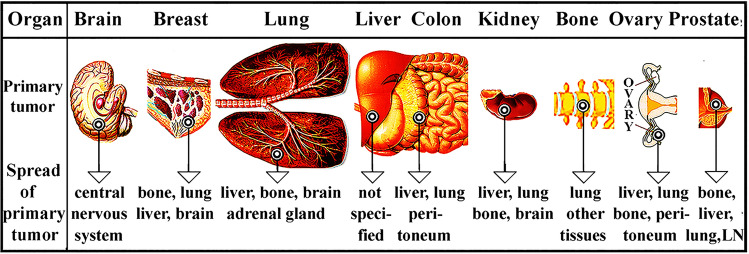
Primary tumours spread to distant organs. The location of the primary tumours in major human organs is indicated by concentric rings

The ^18^FDG-miniPET tumour diagnostic test has been adapted to investigate tumour growth in vivo in local and metastatic rat models. We have confirmed that (*i*) local *s.c*. administration of tumour cells generated local tumours unsuitable to follow the spread of metastasis (*ii*) Intravenous administration of tumour cell cause the unpredictable location of tumour formation, thus not regarded as a reliable metastatic tumour model. (*iii*) Subrenal and liver implantation proved to be suitable to follow the metastatic process in rats [38, 39]. The size range of administered particles should correspond to colloids that are between 1 nm and 10 µm. Viruses, bacteria, small eukaryotic cells (among them yeast, mammalian, and tumour cells), cell remnants, carbon particles (e.g. India ink), and fibrous crystals (e.g. asbestos) belong to this size range. Foreign particles in the body are attacked by white blood cells (subsets of granulocytes, monocytes and lymphocytes). Figure 3 was designed by the author and presened at the World Congress of Breast Cancer, Birmingham, 2015.

**Fig. 4:**
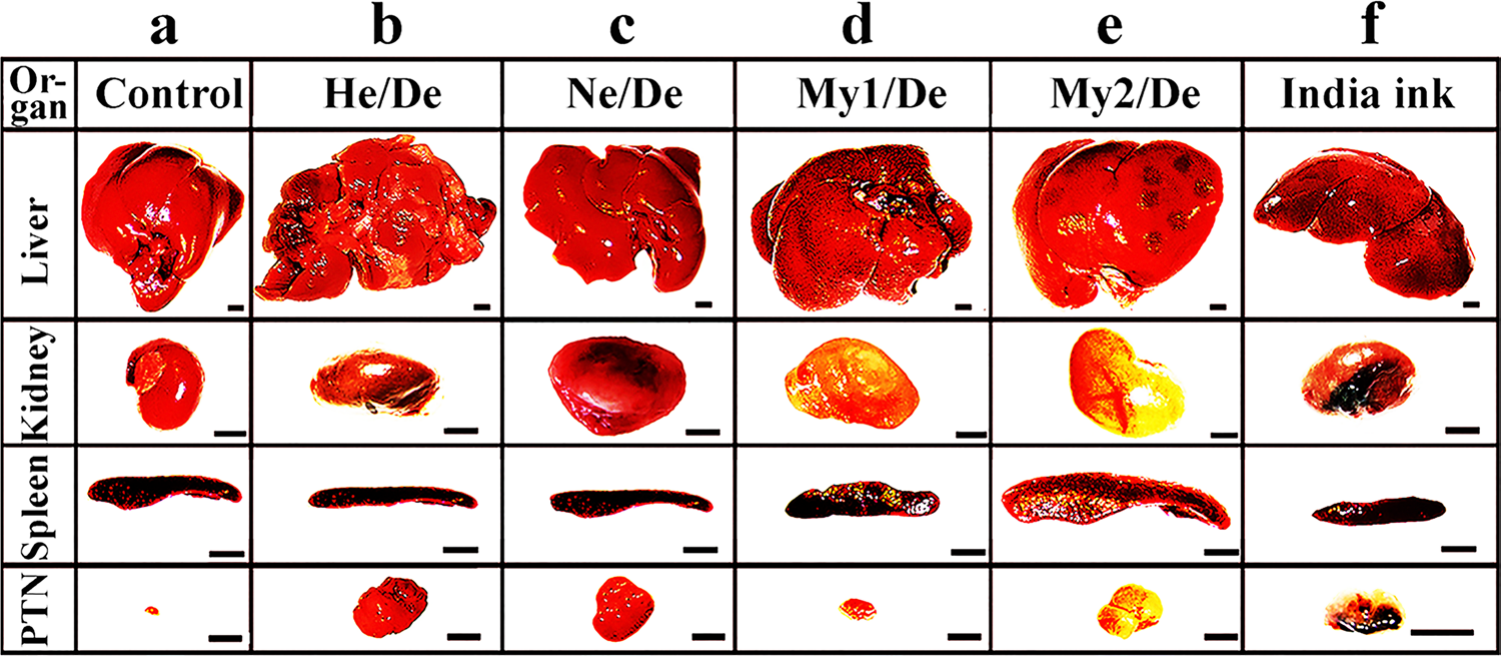
Primary liver tumours spread to other organs. **a** Organs of rats before implanting tumour cells into the liver. **b**-**e** Implantation of tumour or cells inducing abdominal primary tumours or f) colloidal particles. Solid tumour (HeDe, NeDe) cells, (My1De or My2De) (10^6^) were placed under the capsule of the left kidney or the Glisson’s capsule of rats. Two weeks after HeDe or NeDe and four weeks after myeloid leukaemia cell implantation animals were euthanized. The liver, the impacted left kidney, spleen and parathymic lymph nodes (PTNs) were removed surgically *postmortem*. **a** Control: liver, right kidney, spleen and parathymic lymph nodes 14 days after subrenal implantation of saline. **b** Liver hepatocarcinoma formation after HeDe cell (10^6^) implantation under the Glisson’s capsule. Bottom panel: enlargement of parathymic lymph nodes (PTNs). **c** Nephroblastoma formation in the kidney after subrenal implantation of NeDe cells (10^6^) into the liver. Bottom panel: Enlargement of PTNs. **d** Liver, kidney, spleen, PTN enlargement upon subrenal implantation of My1De cells (10^6^). **e** Subrenal implantation of My2De cells (10^6^). **f** Implantation under the renal capsule of 0.1 ml 0.1% India ink and appearance 24 h after administration in the cortical region of parathymic lymph nodes. Abbreviations: LN, lymph node; PTN, parathymic lymph node. Bar, 0.5 cm each

Primary tumours metastasized from organs to other, often distant organs. The exception is the liver, which in this regard is not exactly known where it is projecting tumour cells. To estimate the metastatic potential of liver tumour slices or cells were implanted in the liver’s Glisson pocket and tumour growth is shown in Fig. 4.

The appearance of tumour cells in PTNs was also confirmed by the reimplantation of tumour-bearing parathymic lymph node slices. Reimplantation induced tumour growth under the subrenal capsule. Results corresponded to expectations, after one week of tumour growth there were sufficient number of metastatic cells in PTNs to reinduce primary tumour growth [3].

### Liver, cancer homing centre

**Fig. 5:**
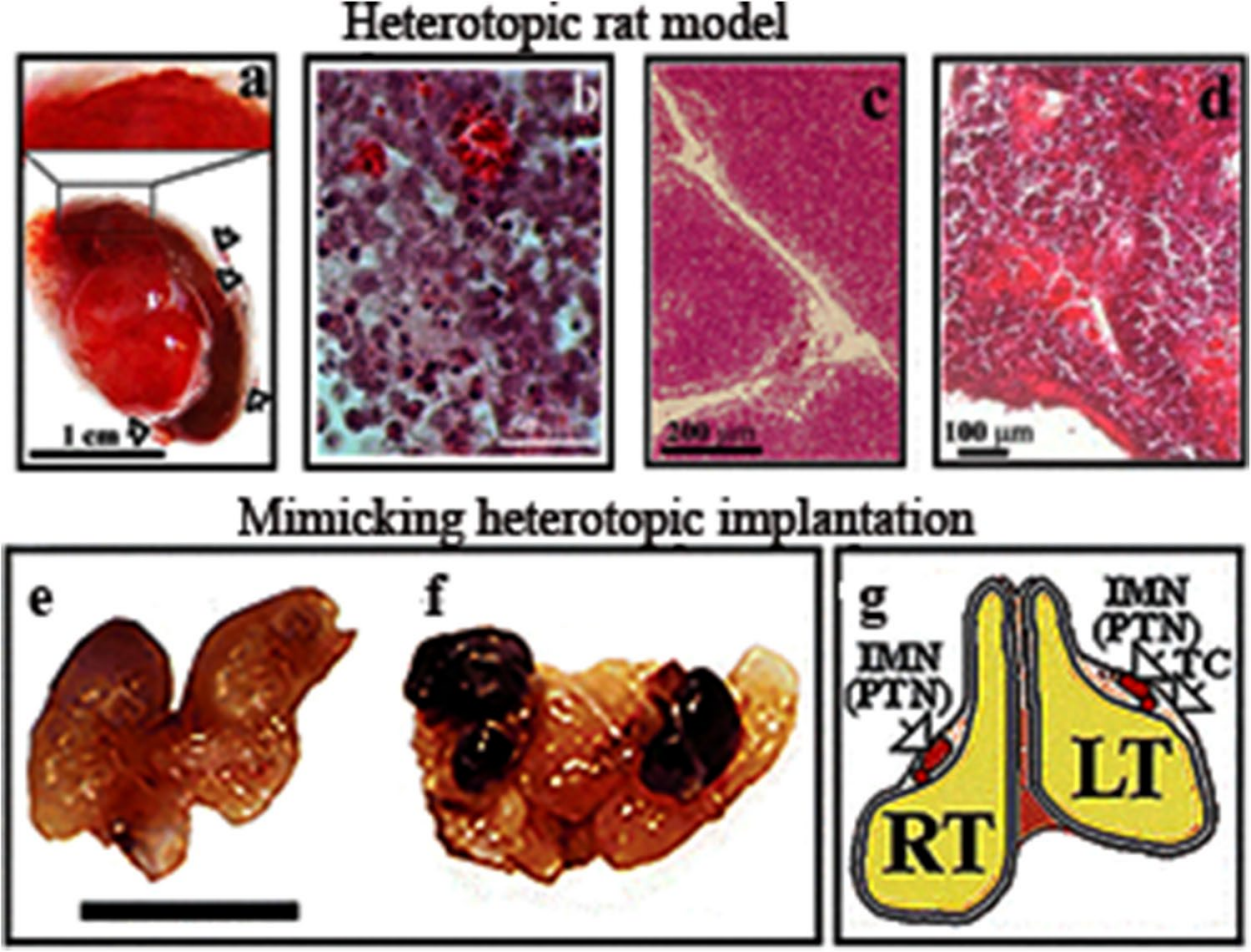
Tumour models to follow the metastatic migration of tumour cells from primary tumours to PTNs *Heterotopic rat model.* Growth and metastatic spread of abdominal primary tumour upon heterotopic implantation of hepatocarcinoma (HeDe) rat tumour cells (10^6^) under the subrenal capsule of rats. **a** Haemorrhagic ruptures in the primary kidney tumour two weeks after implantation. Ruptures are indicated by the arrowheads. The outer part of the primary tumours is seen in the upper right corner of f panel. **b** Hematoxylin–eosin staining of a tissue section near the surface of the tumour. **c**. Hematoxylin–eosin staining of control PTNs.d) Haematoxylin–eosin staining of PTNs two weeks after subrenal implantation of HeDe cells

The organs before the implantation of tumour cells are seen in Fig. 5a. Hepatocellular carcinoma liver tumour cells that metastasized primarily to parathymic lymph nodes and cause the moderate spread to other abdominal organs (kidney, spleen) (Fig. 5b). Nephroblastoma cells implanted in the liver generated tumour and projected metastases to PTNs, impacted liver, but did not induce metastasis in other organs (Fig. 5c). Myeloid leukaemia (My1De, My2De) cells besides causing primary kidney tumour, appeared as parathymic, liver and spleen metastases (Figs. 5d and Fig. 5e. Corresponding to the idea that abdominal tumours spread through the liver regarded as the cancer cell homing centre. India ink implanted under the kidney mimicked metastatic tumour spread, and colloidal carbon particles appeared in the liver but also deposited in the spleen and the more distant parathymic lymph nodes (Fig. 5f). Tumour cell lines have been prepared from rat tumours such as hepatocarcinoma and named Hepatocarcinoma Debreceniensis, abbreviated to He/De, nephroblastoma Debreceniensis (Ne/De), myeloid leukaemia I (My1/De myeloidoid leukaemia 2 (My2-De) from rat tumours [40]. The rat kidney capsule-parathymic lymph node complex as well as the implantation of tumour cells into the liver’s Glisson capsule was proposed for the examination of in vivo primary tumour growth that turned into a metastatic process. Xenograph models, due to the longer periods (weeks) of cancer cell growth were tested after heterotopic and orthotopic implantation of tumour cells into rats and mice. Orthotopic tumours were induced in rats, and tumour cell movement was mimicked after the implantation of India ink colloidal particles. Direct xenografts without cell lines were used to reimplant tumour-bearing slices. The kidney capsule-parathymic lymph node complex was proposed as a new metastatic model for the isolated in vivo examination of secondary lymphatic tumour development [37, 41]. The method of implanting tumour cells under the renal capsule of mice was applied [42] originally for the screening of chemotherapeutic agents [43].

PTNs in rats are located outside the thymus. In mic,e they are harboured inside the thymic capsule [42]. Human parathymic lymph nodes similarly to murine PTNs are hidden inside the thymus. This could be the reason why in the last century only a few reports have dealt with human PTNs. Under the term “parathymic lymph nodes” 62 entries have been found and 1445 items as homologous organs under”internal mammary lymph nodes” in PubMed (as of Apr. 28, 2022). These data show that the clinical research of internal mammary lymph nodes (IMNs) and the study of parathymic lymph nodes inrodentst was done independently without recognizing the structural similarities and functional relationship between them. The tumour development and metastasis formation in these homologous organs need some explanation for the human relevance of animal experiments. Attempts to inoculate human xenografts into rodents, preferentially to immune depleted mice to study metastasis did not result in significant progression. It is even doubted whether such changes caused by human xenografts in mice are characteristic of human cancer.

After the implantation of hepatocarcinoma (HeDe) rat tumour cells under the capsule, the bleeding of the retroperitoneal primary HeDe tumour indicated lagging angiogenesis and ruptures of blood vessels near the surface of the tumour (upper left side in Fig. 5a). The initial infiltration of tumour cells into the healthy tissue gradually turned to an invasion inside the kidney tissue and outward through the peripheral disruptions to the abdominal cavity (Fig. 5). Hematoxylin–eosin staining of tumour sections showed that the primary tumour was more compact and tightly packed with tumour cells outward the primary tumour and released red blood cells and tumour cells through the ruptures seen as red patches. There was a significant difference between the tissue sections of parathymic lymph nodes hematoxylin–eosin staining of control cells (Fig. 5c/), and after 2 weeks of HeDe implantation under the subrenal capsule (Fig. 5d). The invasion of PTNs by the appearance of haemorrhagic patches (Fig. 5d). 

### Mimicking the spread of metastasis (Fig. 5e-f)

Mice were administered *i.p*. India ink. Delivery of India ink particles to mimic tumour cell transmission to PTNs in mice is demonstrated in Fig. 5e-g.

Saline (2 ml) without ink was administered *i.p.* into control C3H mice and healthy thymus was isolated after euthanization (Fig. 5 e).Control murine thymus was isolated after subjecting mice to saline treatment (Fig. 5f). PTNswere filled with colloid 24 h after *i.p.* injection of India ink. Four black nodes show the accumulation of ink particles in the PTNs of thymic tissue. Bar: 0.5 cm. g) Schematic view of the thymus. RT, right lobe of the thymus; LT, left thymic lobe; IMN, internal mammary lymph node identical with PTN, parahymic lymph node; TC, thymic capsule. With permission [40]. (Presented at the Congress on Breast Cancer. Banfalvi G. Origin of breast cancer metastasis. 2015, Birmingham, UK).

After 48 h of *i.p.* delivery of India in,k the parathymic lymph nodes of the murine thymus were packed with the ink. The ink particles accumulated in two larger and two smaller nodes (Fig. 5). In earlier work Lynn described two to four nodes lying immediately behind the murine thymus [44]. Figure 5f confirmed that the four PTNs covered by the thymic capsule were not harboured inside the PTNs of rats extruded and could be isolated by microsurgery. The microsurgical removal of these tiny lymph nodes will be of medical importance, especially when the spread of metastatic migration to others *e.g.* mammary lymph nodes should be avoided and when the gaining of time would be critical for a combined therapy to extend the life of patients.

The surgical removal of PTNs could be important in thymus transplantation to avoid transfusion-associated graft *versus* host disease. The secure microsurgery of murine PTNs needs improvement, whereasthoset-mortemem removal of clearly distinguishable rat PTNs especially after metastatic enlargement was rutinely performed. As far as cancer cell homing is concerned, liver is the second most frequently impacted organ worldwide [45].

### Human aspects of metastasis

By analogy human tumours in the lymph nodes of the thoracic cavity are likely to accumulate in the parathymic lymph nodes and cause metastasis not only in the internal mammary lymph nodes but could spread to the mammary lymph nodes. The review of human aspects of metastatic spread is based on animal experiments using tumour cell lines: Hepatocarcinoma (HeDe) and mesoblastic nephroma (NeDe) solid tumours [3] as well as myelomonocytic leukaemia lines [39, 46]. The advantage of indirect xenografts from cell lines, over original tumour explants without a cell line intermediary obtained from tumour-bearing rats was that direct tumour implantation into rodents could be avoided. The reproducible implantation of exact number of cells from tumour cell lines allowed the tracing of primary tumour development and metastatic spread in recipient inbred Fischer 344 rats of the same litter. Clinical oncology is not only supported by, but depends on strictly controlled animal experiments that provide a suitable means to follow abdominal tumour growth and its escalation to thoracal and mammary metastatic lymph nodes.

Despite their mutagenic impact,’double-edged’ molecules could help those patients who are older than their fertility age, and the anticancer treatment could be more important than the long-term mutagenic affect the development of which could take years or decades. Two groups of patients could be particularly interested in the treatment with Janus-faced anticancer agents. The incidence of breast cancer, the most common malignancy in women increases with age, with the patients diagnosed after menopause. In about 15–25% of cases, patients are premenopausal at the time of diagnosis of cancer, and about 7% are below the age of 40 [47].

The treatment of postmenopausal breast cancer patients with Janus-faced anticancer cytotoxins is preferentially recommended, unlike prostate patients. Although the decline in sperm count, and quality (morphology, motility, fragmentation of DNA) is measurable but men typically never stop producing sperm, thus the fertility age is less important than in women.

Immunocompromised mouse strains allowed researchers to implant human tumour cells into mice without the risk of cell-mediated immunological tissue rejection but are not useful for studying immune-mediated responses to malignancy [48].

As abdominal tumour development and its appearance in distant thoracal sentinel lymph nodes and regional spread of gynaecological or other malignancies [48–50] is not well known, we have followed the metastatic activities of organs after in vivo implants have been established.

The growth pattern of primary abdominal tumours in rodents (rats, mice) exhibited markedly similar features: peripheral ruptures of blood vessels near the surface of the primary tumour, shedding tumour cells into the abdominal cavity, crossing the stomata of the diaphragm and causing metastatic spread in thoracal, primarily parathymic lymph nodes (PTNs). Similarly, the intraperitoneal administration of Indian ink colloidal particles bypassed the distributing vascular route and tumour cells accumulated in PTNs. The thoracic duct drains into the systemic blood circulation at the angle of the left subclavian and internal jugular veins. At this point, the tumour spread becomes generalized and metastatic foci can develop wherever the circulation of blood is obstructed.

**Fig. 6:**
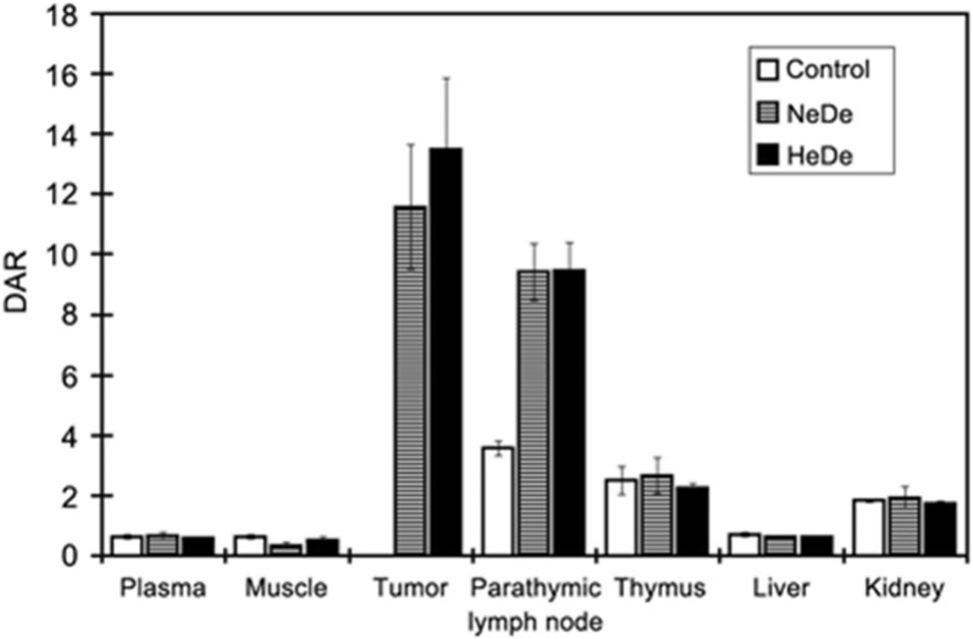
Tissue distribution of radioactivity after *i.v.* administration of ^18^FDG in Ne/De and He/De tumour-bearing rats. Metastatic potential of Ne/De and He/De cells, plasma, muscle, tumour, paratumourlymph nodes, thymus, liver and kidney expressed as differential absorption ratio (DAR). With permission [37]

The metastatic spread of tumour cells was supported by ^18^FDG-PET examinations*.* High differential absorption ratios (DAR values) as”hot spots” as well as miniPET images of rats served as potential metastatic indicators for the early diagnosis of micrometastases before the upper thoracal lymph nodes would have been affected (Fig. 6).

It is forbidden to inject live cancer cells into patients since the highly unethical practice of injecting HeLa tumour cells into elderly patients [51]. Basic and translational breast cancer research relies exclusively on experimental animal models. It was suggested that results obtained by animal models can be extrapolated to human medicine as a mammal is sufficiently like humans in its anatomy and physiology (https://www.merriamwebster.com/dictionary/animal%20model). Rodents, especially mice and rats, are the most popular animals for breast cancer research [52].

**Fig. 7:**
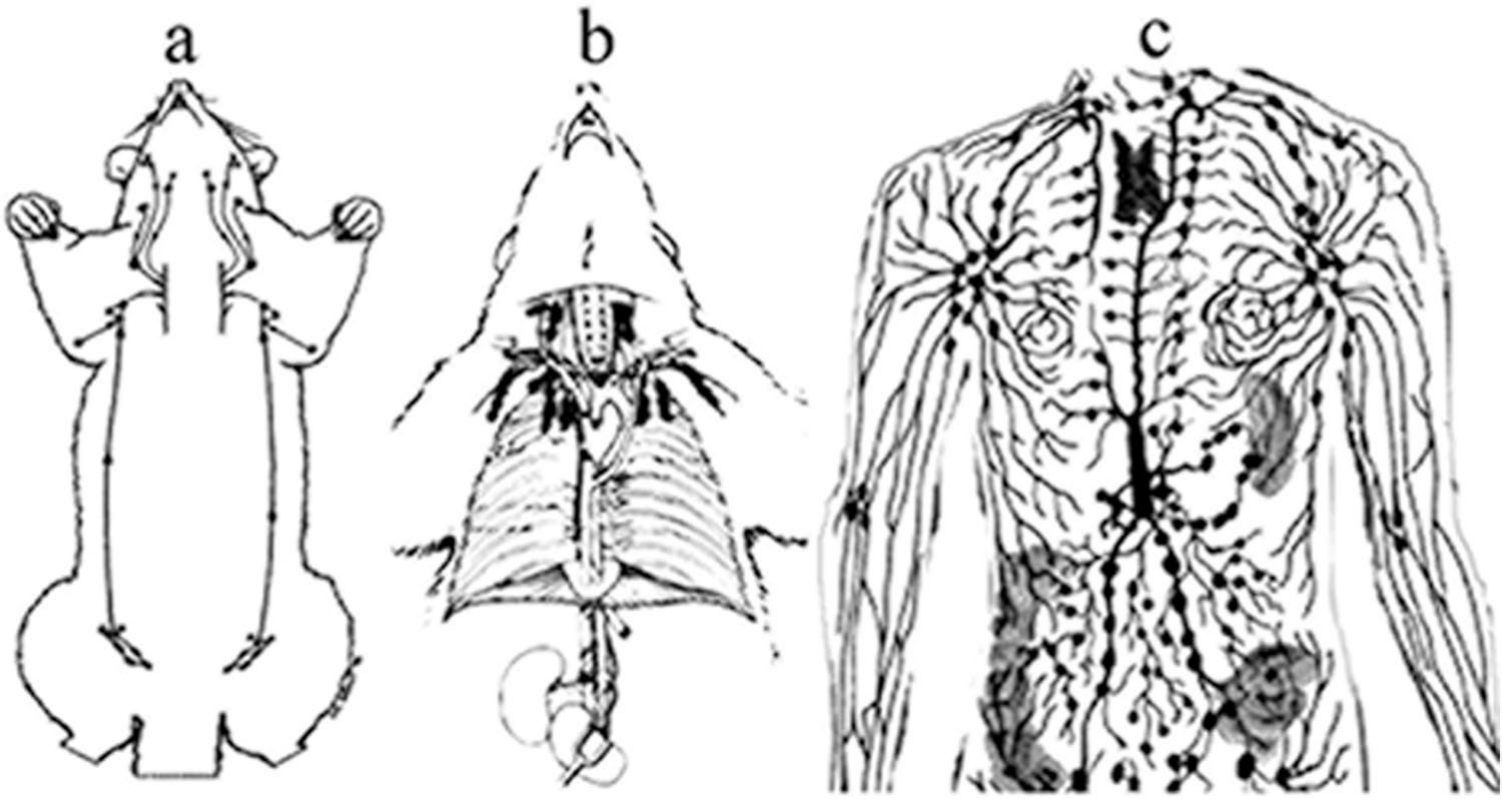
Comparison of lymph nodes in the thoracic region. **a** The low number and small size of thoracic lymph nodes explain why opossums are inefficient against tuberculosis. **b** The lymphatic system of rats is resistant to tubercle bacilli. **c** The lymphatic system of the human thorax is more resistant to tuberculosis than marsupials but less developed than the upper thoracal lymph nodes of rats. With permission [56]

Connections among thoracic and mammary lymph nodes are poorly understood but could explain the metastatic differences not only between marsupials and mammals but also among mammalian species. The chain of human thoracic lymph nodes that continues in the axillary region suggests that in man and modified higher in mammals, these nodes are in a closer relationship than in marsupials (Fig. 7a) similarly to lower mammals such as rodents (Fig. 7b). Regarding human lymphatic spread associations of breast lymph nodes exist between parasternal and thoracic lymphatic vessels, abdominal cancer cells tend to spread along lymphatic passages, main lymphatic drainage of the breast takes place via axillary nodes, and the remaining part of drainage through parasternal nodes, until the haptic blockade could be redirected to tumour spread, and lymph carrying cancer cells drain to the opposite direction (Fig. 7c). The direct lymphatic connection between abdominal tumour cells and thoracal lymph nodes suggests that early detection of micrometastases in thoral lymph nodes could provide enough time for tumour therapy and prevent breast cancer development.

Experiments related to animal models [52], veterinary and clinical medicine [53], comparative oncology [54, 55], and cancer drug resistance [56, 57], significantly contributed to the development of metastasis research and prepared the way for alternate sources of anticancer drug therapy.

### Janus-faced cytotoxins against metastasis

Mycotoxins are promising candidates but not yet applied as medicine. One of the noncerns is the high resistance of tumour cells toward chemotherapy [56] aggravated by the multidrug resistance representing a further impediment to the chemotherapy [57]. Nevertheless, the high cytotoxicity of pro-apoptotic antifungal mycotoxins represents a promising tool against the spread of metastatic tumours. Promising examples of future hopes are those mycotoxins that are listed in Table 1. These mycotoxins possess both the apoptotic and the antitumour potential such as alternariol causing apoptosis in murine hepatoma cells [58]. Similarly, it was found that several other mycotoxins including beauvericin [59–63], cytochalasins and derivatives [63–67], deoxynivalenol [68], mixed with mycotoxins (T-2 and HT-2) [69], deoxynivalenol and fumonisin [85], enniatins A1, B and B1 [86], rubratoxin A [87], rubratoxin B [88], safratoxin H [89] exerted an apoptotic effect on tumour cells. The cytotoxicity of trichothecene type (T-2 and HT-2), type B (deoxynivalenol, nivalenol) and type D (satratoxin G and H) is towards different cell lines quite similar, within 4 nM and 5 µM IC50 concentration range, suggesting that the antitumor potential of trichothecenes has been underestimated [90].

**Table 1:** Apoptotic and antitumour mycotoxins

Mycotoxins	Apoptotic effect on	References
Alternariol	Murine hepatoma cells	[58]
Beauvericin	Lymphoblastic leukaemia	[59–63]
Cytochalasins and drivatives	Cancer cells, actin	[63–66]
Deepoxy- deoxynivalenol	Bovine ovarian theca cells	[67]
Deoxynivalenol	Human cells	[68]
Mixed mycotoxins (T-2 and HT-2)	Primary hepatocytes in broilers	[69]
Mycophenolates	Pancreatic tumours, sarcoidosis	[70–84]
Nivalenol, deoxynivalenol and fumonisin	Human erythroleukaemia cell line	[85]
Enniatins A1, B and B1	Hepatoma cells	[86]
Rubratoxin A	Inhibits protein phosphatase 2A and suppresses cancer metastasis	[87]
Rubratoxin B	Antitumor effect	[88]
Satratoxin H	Generated ROS in PC12 cells	[89]

The metastatic development of peritoneal and retroperitoneal primary tumours to parathymic lymph node metastasis reflects a general mechanism of tumour spread starting with the effusion of tumour cells of the primary tumour through the disrupted veins, crossing the diaphragm entering thoracal lymph nodes and potential progression into mammary lymph nodes.The data of Table 1 suggest that there could be many more antineoplastic mycotoxins that cause apoptosis and could be candidates in the fight against metastasis.

The growth of perioneal and retroperitoneal tumors developing into parathymic lymph node metastasis reflects a general mechanism of tumour spread starting with the effusion of tumour cells of the primary tumour through the disrupted veins, crossing the diaphragm entering thoracal lymph nodes and potential progression into mammary lymph nodes.

### Blood-borne and lymphatic phases of metastasis

The human aspects of metastatic spread are based on animal experiments using tumour cell lines established from chemically induced rat tumours. The advantage of indirect xenografts created from cell lines, over original tumour explants without a cell line obtained from tumour-bearing rats was that direct tumour implantation into rodents could be avoided. The abdominal-thoracal route suggests a novel lymphatic connection between abdominal tumours and mammary lymph nodes and could provide the missing link between the abdominal tumours and breast cancer. Thoracal lymph carrying tumor cells can drain the opposite direction, when lymphatic blockade redirects tumour spread. The direct lymphatic connection between the diaphragm and mammary lymph nodes was published in Gray’s human anatomy [91]. Unfortunately, the close relationship between internal mammary lymph nodes (IMNs) and parathymic lymph nodes as well as the direct lymphatic route from the diaphragm to IMNs remained unnoticed.

**Fig. 8:**
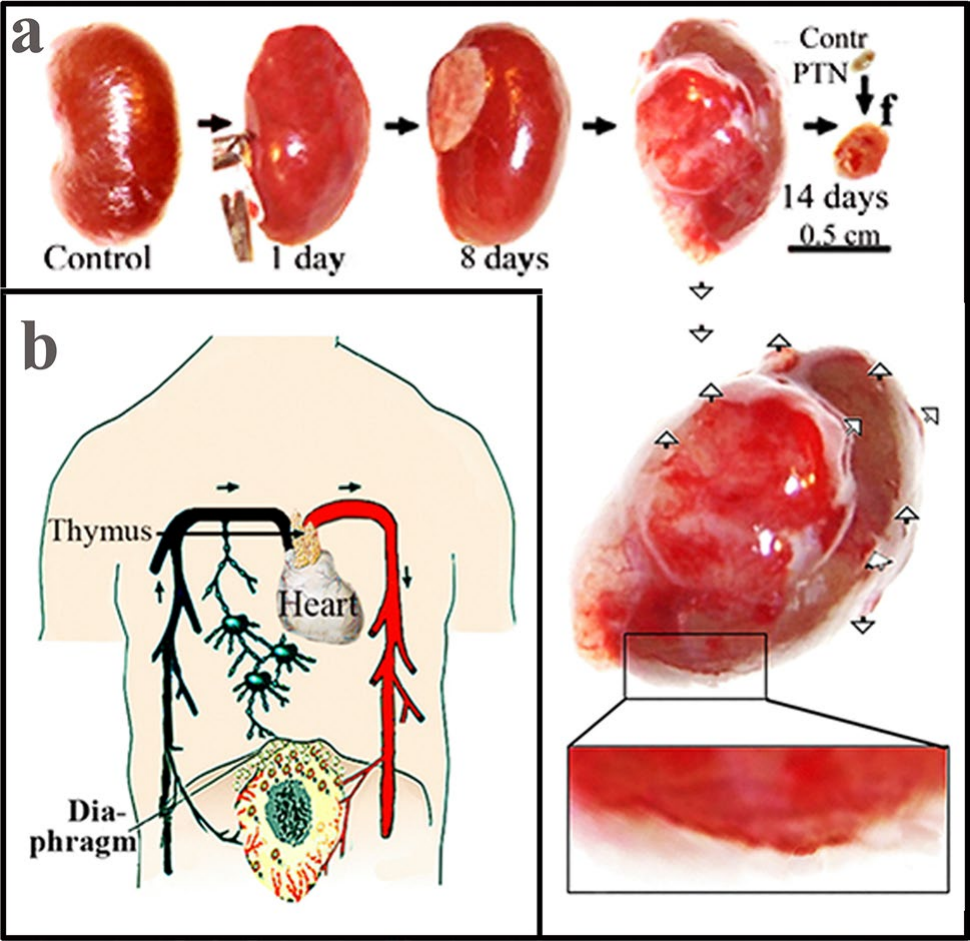
Blood-borne and lymphatic phases of metastasis. Phase (1). Hematogeneous growth of primary tumour cells (**a**). Phase (2) Hematogeneous growth of primary tumour and release of tumour cells into the abdominal cavity, and passage through the diaphragm (**b**). Phase (3) Lymphatic migration of tumour cells from the thoracal capillaries to the blood circulation at the left subclavian and internal jugular veins (**b**). Phase (4) Generalization of metastasis through blood circulation (**b**)

Based on the structural analogy between mammalian lymphatic systems and functional similarities in tumour development and spread, it is reasonable to assume that tumour cells shredded the human abdomen cross the diaphragm and deposit in internal mammary lymph nodes (IMNs) underneath the chest wall, similarly to the accumulation of tumour cells in the parathymic lymph nodes (PTNs) in rats. Experimental tumour models helped to summarize the metastatic hypothesis and subdivide it into four phases. Abdominal primary tumour formation—phase 1. Growth of abdominal primary tumour, with necrotic cells inside the tumour (black) and reduced angiogenesis outward causing the release of tumour cells through the, disrupted blood vessels. Phase 2 passage of tumor cells through the diaphragm (Fig. 8b) Tumour cells cross the diaphragm through the three major pores and enter the chest. Phase 3: lymphatic phase (Fig, 8b). Tumour cells are taken up by the lymphatic capillaries in the thorax. Lymphatic capillaries are larger in diameter than blood capillaries. The lymph capillaries are between10 and 60 µm and permit interstitial fluid and tumour cells to flow in but not out. The lymphatic phase ends when the chyle flowing from the thoracic duct into the systemic blood circulation at the angle of the left subclavian and internal jugular veins. Phase 4: Generalization of metastasis—2nd vascular phase. The tumour cells return to the blood circulation cells and can spread everywhere in the body where circulation is hindered and the soil is fertile enough for cell to settle down and suitable for metastatic growth.

Lymphatic vessels of the diaphragm follow the course of their corresponding vessels and terminate not only in the anterior and posterior mediastinal lymphatics but more importantly in the intercostal and internal mammary lymph nodes of the laboratory rat [92]. This route drains the abdominal tumour cells via the lymphatic vessels from the diaphragm directly to the mammary glands. The neglected lymphatic connection between abdominal primary tumours and mammary lymph nodes could provide the missing link between the abdominal tumour growth and metastases in the mammary gland. Equally important is the observation that the lymph carrying cancer cells can drain to the opposite direction, and the lymphatic blockade can redirect tumour spread (www.oucom.ohiou.edu/dbms-witmer/downloads/2012-04-24_dashner_rpac-breastanatomy.pdf)

Improved reproducibility, larger than murine body size, well-characterized and human-like physiology, made the rat tumour model indispensable for metastatic research. Another likely reason why human mammary lymph nodes in the development of breast cancer metastasis cannot be used is the hidden location of IMNs inside the thymic capsule. The rat metastatic model helped to recognize the spread of metastasis through the enlargement of PTNs, whereas the accumulation of tumour cells in murine and human PTNs enclaved in the thymic tissue remained undetectable. In addition to the findings of experimental metastasis, clinical data support the hypothesis that human breast cancer may originate from primary tumours.

### Anatomical and clinical evidence for the metastatic spread of abdominal tumours

The tumour development in rodent models is supported by clinical and anatomical evidence suggesting that a considerable proportion of metastases in the breast may originate from internal mammary (*alias* parathymic) lymph nodes, or spread via direct connections between the abdomen and the thorax in a lymphatic rather than a vascular manner. Anatomical evidence for the lymphatic connection between the diaphragm and mammary lymph nodes was provided [93].

Clinical investigations related to the lymphatic drainage of the breast reported the possibility of bidirectional movement to the axillary lymph nodes and drainage from the internal mammary lymph nodes [94, 95]. The importance of the metastatic spread is supported by the following clinical observations: In almost all breast cancer patients the internal mammary lymph node tumour was the forerunner of the metastatic disease [96]. In cases of carcinomata without axillary involvement, there was mortality from recurrence of about 25% in the first five years after breast operation [97] leading to the conclusion that if the axillary lymphatics were the only path by which carcinoma cells could escape from the breast, this high mortality would be inexplicable. The clinical cases suggested that internal mammary glands were invaded before cancer cells have reached the axilla [98]. Clinical evidence indicated a relatively high incidence (~ 25%) of secondary tumour spread to the breast [98]. In a Canadian study, the radiation therapy involved 1,000 women who were subjected to internal mammary node radiation. It was feared that if cancer has spread to these lymph nodes, it could have increased the risk of uncommon heart and lung problems as the tissues could have been unintentionally exposed to radiation (http://www.breastcancer.org/research-news/20110912). However, advances in radiation therapy technology make it unlikely that healthy nearby tissues would be exposed. Moreover, advances in diagnostic and surgical skills prove that IMN biopsy and removal can be performed safely without complications [98]. Another study attempted to confirm IMN metastasis pathologically and to improve the survival rate through simple surgical removal and additional radiotherapy. Although most of the lymph nodes and intercostal fat harboring metastasis were removed, some lymph nodes may have remained behind the sternum and ribs [99]. Studies in this respect have shown that axillary nodal dissection alone resulted in understating up to 16% of women with negative findings from exploration of axillary nharbouredbored occult metastases to the internal mammary nodes [100]. These observations confirmed the notion that IMN metastasis is likelproceedeceed axillary lymph node metastasis and support the metastatic view of breast cancer, namely that breast cancer is not exclusively a primary tumour, but can be a metastasis as well [55, 101].

## Conclusion

It is assumed that in mammals the tumour cells shed by primary tumours through the disrupted blood vessels of the primary tumour to the peritoneal and retroperitoneal cavities cross the diaphragm and are taken up by the thoracal lymph vessels, carried to and accumulated in the internal mammary lymph nodes. Several arguments have been listed supporting the idea that tumour-bearing lymph nodes among them mammary lymph nodes are not primary tumours, but metastases [102]. This review by comparing the lymphatic spread of abdominal tumours in different mammals concludes that:

- associations of breast lymph nodes exist between parasternal and thoracic lymphatic vessels,
- abdominal cancer cells tend to spread along lymphatic passages,
- main lymphatic drainage of the breast (75%) is via axillary nodes, but the remaining drainage (25%) is through parasternal nodes,
- unilateral lymphatic blockade may redirect tumour spread,
- lymph that is carrying cancer cells can also dinin to opposite te direction www.oucom.ohiou.edu/dbms-witmer/downloads/2012-04-24_dashner_rpac-breastanatomy.pdf.

The metastatic spread of tumour cells was supported by ^18^FDG-PET examinations. High DAR values as”hot spots” and miniPET images of rats served as potential metastatic indicators for the early diagnosis of micrometastases before other organs (e.g. mammary lymph nodes) would have been affected [37]. Our view of progressive and stepwise metastasis is supported by others who found that breast cancer cases with patients upon internal mammary lymph node recurrence or local metastasis is still having a good prognosis [101]. Results call attention to the early diagnosis of abdominal tumour cell and thoracal micrometastases before the last defence line represented by PTNs has been exhausted and before the lymph would return to the blood circulation and cause the generalization of the metastatic process. Early detection of micrometastases in thoracal thymphlymph is recommended to prevent mammary breast cancer development and to gain time for tumour therapy. As far as the treatment of the metastatic process is concerned antifungal agents have been included’Janus-faced’ mycotoxins, and aminoglycoside molecules among them the most efficient minor fraction gentamicin B1 [8]. The antifungal potential of mycotoxins was comparable to the classical antifungal drugs. The double-edged sword effect of antineoplastic mycotoxins affecting eukaryotic cells due to the broad range of structural and functional groups, of these cytotoxins contributed to their different types of classification. The drastic anticancer potential of the following mycotoxins deserve further tests: ergotamine, cyclopiazonic acid, T-2 toxin, satratoxin H, alternariol, pseurotin, synerazol, rubratoxin, beauvericin, enniatin, tenuazonic acid, cytochalasin B, cytochalasin C, MT81[40] among the ever increasing number of new compounds [22].

The incidence of breast cancer, the most common malignancy in women increases with age, with the majority of patients diagnosed after menopause. In about 15–25% of cases, patients are premenopausal at the time of diagnosis of cancer, and about 7% are below the age of 40 [40]. The treatment of postmenopausal breast cancer patients with Janus-faced anticancer cytotoxins is preferentially recommended, unlike prostate patients. The utilization of apoptotic effects against tumour growth by agents that, at the same time, induce mutations may be of ethical concern. Nevertheless, despite the mutagenic impact of’double-edged’ molecules could be helpful for patients who are over their fertility age, and the prevention of breast cancer is likely to be more important than the long-term mutagenic effect that may impact their health in years or decades yet to come.

## Data Availability

The authors confirm that the data supporting the findings of this study are available within the article. Supplementary material associated with this article can be found at HTTPS: HTTP.
